# Natural Products Against *Mycoplasma gallisepticum*: Emerging Alternatives to Combat Antimicrobial Resistance

**DOI:** 10.3390/microorganisms14061222

**Published:** 2026-05-28

**Authors:** Rong Xi, Ban Li, Yue Wu, Chengbo Wen, Yunchen Zhou, Zhiyong Wu, Dexian Zhang, Jichang Li

**Affiliations:** 1 School of Animal Science and Technology, Foshan University, Foshan 528231, China; 17866927857@163.com (R.X.); a13138288071@163.com (B.L.); wuyue12262022@163.com (Y.W.); bwcheng0023@163.com (C.W.); zhouyunchen2025@126.com (Y.Z.); 2 College of Veterinary Medicine, Northeast Agricultural University, Harbin 150030, China; wuzhiyong@neau.edu.cn

**Keywords:** *Mycoplasma gallisepticum*, antimicrobial resistance, natural products, poultry

## Abstract

Antimicrobial resistance in *Mycoplasma gallisepticum* (MG), a primary causative agent of chronic respiratory disease in poultry, has reached alarming levels, underscoring the urgent need for alternative strategies. Natural products have emerged as promising candidates owing to their multi-target mechanisms of action. This review synthesizes current evidence on natural anti-MG agents, critically appraising their in vitro and in vivo efficacy, molecular mechanisms, and translational potential. A mechanistic taxonomy is proposed for distinguishing direct pathogen-directed mechanisms (membrane disruption, adhesion inhibition, virulence factor neutralization) from indirect host-directed mechanisms, notably NF-κB/MAPK pathway modulation and gut–lung axis immunoregulation. Emphasis is placed on anti-infective polypharmacology, exemplified by luteolin’s dual inhibition of the TatD virulence factor and host inflammatory cascades. The gut–lung axis represents a novel therapeutic frontier, with *Bacillus subtilis* KC1 controlling respiratory mycoplasmosis through intestinal microbiome remodeling and systemic AhR activation. Despite encouraging efficacy data, critical knowledge gaps persist, including a scarcity of rigorous in vivo trials under commercial conditions, incomplete mechanistic characterization, and challenges in standardizing complex natural product formulations. Natural products are best positioned not as wholesale antibiotic replacements but as integral components of integrated, antibiotic-sparing strategies aligned with antimicrobial stewardship and One Health principles.

## 1. Introduction

*Mycoplasma gallisepticum* (MG) is widely recognized as one of the most economically destructive pathogens affecting the global poultry industry [1,2]. As the primary etiological agent of chronic respiratory disease (CRD) in chickens and infectious sinusitis in turkeys, MG infection leads to reduced weight gain, impaired feed conversion, decreased egg production, elevated mortality, and increased carcass condemnation at slaughter [3,4]. The pathogen’s unique biological characteristics, including its minute size (0.2–0.8 μm), the absence of a peptidoglycan cell wall, and the possession of a cholesterol-rich triple-layered plasma membrane, confer intrinsic resistance to β-lactams, cephalosporins, and glycopeptides while contributing to its pleomorphic and motile nature [5].

The economic burden imposed by MG is further amplified by its capacity for synergistic co-infections [6]. MG-induced damage to the respiratory epithelium, particularly the disruption of mucociliary clearance, creates a permissive environment for secondary bacterial invaders such as *Escherichia coli* (*E. coli*), resulting in severe airsacculitis and polyserositis [7]. Co-infections with respiratory viruses, including Newcastle disease virus and infectious bronchitis virus, similarly exacerbate clinical severity and complicate therapeutic management [8,9]. Critically, antimicrobial therapy does not eliminate the chronic infection; persistently infected birds remain reservoirs for both horizontal and vertical transmission, perpetuating infection cycles within and between flocks [10].

For decades, the control of MG has depended upon the administration of antimicrobial agents, with macrolides (tylosin, tilmicosin, tylvalosin), tetracyclines (doxycycline, oxytetracycline), and fluoroquinolones (enrofloxacin, danofloxacin), and pleuromutilins (tiamulin) representing the predominant drug classes employed in poultry medicine [11]. The widespread and frequently indiscriminate use of these antibiotics has been paralleled by a persistent escalation in antimicrobial resistance. Recent surveillance data reveal concerning resistance rates: one study of Tunisian field isolates reported that 87.5% were resistant to tilmicosin and 68.8% to tylosin. Furthermore, high levels of resistance are increasingly recognized as a significant problem in Southeast Asia [12,13]. Similar trends of declining susceptibility have been reported across multiple geographic regions, raising serious concerns regarding the long-term viability of current therapeutic regimens [14,15,16].

The emergence of multidrug-resistant (MDR) MG strains, combined with growing regulatory restrictions on antibiotic use in food-producing animals and mounting consumer demand for antibiotic-free poultry products, has created an urgent impetus for the development of alternative control strategies. Natural products, including medicinal plant extracts, essential oils, and bioactive compounds from algae and other sources, have attracted considerable research attention as potential antibiotic alternatives [17,18]. These agents frequently possess complex, multi-target mechanisms of action that may circumvent existing resistance pathways, while also offering ancillary benefits such as antibacterial, immunostimulation, anti-inflammatory activity, growth promotion and improved digestive enzymes, and gut barrier functions [19].

This review aims to provide a comprehensive synthesis of the current evidence regarding natural products with anti-MG activity. We examine the major classes of natural compounds investigated to date, critically appraise the available in vitro and in vivo efficacy data, explore the molecular mechanisms underlying their therapeutic effects, and identify key knowledge gaps and future research priorities. By situating natural products within the broader context of the antimicrobial resistance (AMR) crisis, this review seeks to inform the development of sustainable, antibiotic-sparing strategies for MG control in commercial poultry production.

## 2. The Antimicrobial Resistance Crisis in MG

### 2.1. Current Status of Antibiotic Resistance

Recent surveillance data paint a concerning picture of resistance prevalence. The Tunisian study that serves as a foundational reference for much contemporary natural product research documented 87.5% resistance to tilmicosin and 68.75% resistance to tylosin among 64 field isolates [12]. In Egypt, multiple investigations have confirmed declining susceptibility to formerly first-line agents, with some isolates demonstrating MIC values exceeding clinically achievable concentrations [20]. These findings are not geographically isolated, and analogous trends have been reported from Asia, Europe, and the Americas, indicating that AMR among avian mycoplasmas is a genuinely global phenomenon [21,22,23].

The acquisition of antimicrobial resistance in MG is almost exclusively attributable to chromosomal mutations in genes encoding antibiotic target sites, as MG has not been demonstrated to harbor horizontally acquired resistance determinants such as plasmids or conjugative transposons [11]. The most clinically significant mutational events include A2058G and A2059G transitions in domain V of the 23S rRNA, which confer high-level resistance to macrolides [24]; Ser83Ile/Asn/Arg substitutions in GyrA, which reduce fluoroquinolone binding affinity [25]; and mutations within the *parC* gene encoding topoisomerase IV, which act synergistically with *gyrA* alterations to produce clinically relevant high-level fluoroquinolone resistance [26]. While the mutations involved in antimicrobial resistance (AMR) in MG are listed in Table 1, it should be noted that additional mechanisms beyond mutational changes may also contribute to AMR in MG, including biofilm formation, active efflux, and enzymatic drug inactivation [27].

### 2.2. Implications for Poultry Production and One Health

The consequences of AMR in MG extend well beyond the clinical outcome of individual flocks. At the production level, treatment failures lead to protracted outbreaks, increased mortality, and greater economic losses. The subsequent escalation of antibiotic usage, whether through higher doses, prolonged treatment courses, or the deployment of last-resort drugs, intensifies selection pressure, further accelerating resistance development [24,32]. Furthermore, antibiotic misuse disrupts the normal microbiota, compromises host immunity, and elevates the risk of drug residues entering the human food chain [33,34].

From a One Health perspective, the use of macrolides and fluoroquinolones in poultry has direct implications for human medicine. This concern extends beyond the treatment of zoonotic pathogens such as *Campylobacter* and *Salmonella*, as the acquisition of resistance by ESKAPE organisms and the supply of resistance determinants to the environmental resistome represent equally critical threats [35,36]. The co-selection of resistance determinants in both commensal and pathogenic bacteria within the poultry production environment underscores the interconnected nature of animal and human health [37]. These considerations have strengthened the rationale for exploring natural alternatives that do not exhibit cross-resistance with conventional antibiotics and may therefore reduce overall selection pressure within the agricultural ecosystem.

## 3. Natural Products with Anti-MG Activity: Current Evidence

The control of MG infection in poultry has catalyzed an extraordinary diversification of therapeutic strategies, spanning from crude natural extracts to precisely designed synthetic molecules, and from live microorganisms to host-encoded regulatory RNAs. A systematic analysis of the agents reviewed herein reveals a field in transition, characterized by a progressive shift away from conventional antibiotic monotherapy toward multi-modal, host-inclusive approaches.

### 3.1. Tiamulin and Beyond: A Continuum of Anti-MG Therapeutics

The anti-MG therapeutic landscape can be conceptualized as a developmental continuum anchored at one end by validated clinical benchmarks and extending at the other toward emerging therapeutic frontiers. Tiamulin, the first-generation pleuromutilin, occupies the position of the established standard-of-care, serving as the indispensable positive control against which the efficacy of virtually all investigational agents is measured. Its repeated use as a comparator has provided the field with a consistent and clinically meaningful efficacy threshold, and the demonstration that certain natural products, notably fermented wheat germ extract and quercetin, can achieve therapeutic parity with tiamulin represents one of the most significant collective findings of the reviewed literature [38,39]. This parity challenges the assumption of inherent antibiotic superiority and provides a robust evidentiary foundation for the development of non-antibiotic alternatives.

Moving along the continuum, the novel semi-synthetic pleuromutilin derivatives amphenmulin and p-furoylamphenmulin represent the refinement and optimization of the established ribosomal inhibition paradigm. These agents achieve extraordinary gains in intrinsic potency, with minimum inhibitory concentrations (MICs) of 0.0039 μg/mL and 0.00195 μg/mL, respectively, values orders of magnitude lower than those of their first-generation predecessors and the vast majority of natural alternatives [40,41]. Their comprehensive pharmacokinetic/pharmacodynamic (PK/PD) characterization, including the generation of defined therapeutic targets through Sigmoid Emax modeling, positions them as the most translationally advanced candidates from a clinical dosing perspective. However, their mechanism, ribosomal protein synthesis inhibition, is susceptible to well-characterized resistance determinants, including mutations in the 23S rRNA gene and the *vga* and *lsa* efflux pump genes, underscoring the inherent limitations of single-target, direct-acting antimicrobial strategies [42].

The nanoparticle–antibiotic combination strategy, exemplified by Tylvalson (ECO Animal Health, London, UK) co-administered with zinc oxide nanoparticles (ZnO-NPs), introduces a complementary innovation pathway that enhances the performance of existing agents through advanced delivery systems. The combined aivlosin/ZnO-NPs regimen was demonstrated to be the most successful therapeutic approach in a polymicrobial MG-*Ornithobacterium rhinotracheale* co-infection model, significantly reducing clinical signs, mortality rates, and histopathological lesions, while also decreasing aivlosin residue levels in edible tissues, a dual benefit addressing both therapeutic efficacy and food safety concerns [43].

### 3.2. The Rise in Host-Directed and Multi-Target Natural Products

The most conspicuous trend in the anti-MG field is the ascendancy of purified phytochemicals as validated therapeutic candidates. These compounds collectively demonstrate that a host-directed, anti-inflammatory therapeutic strategy can achieve outcomes comparable or equivalent to those of conventional antibiotics. A comparative analysis of these agents reveals a hierarchy of pharmacological validation and translational readiness.

Baicalin emerges as the most comprehensively characterized phytochemical, distinguished by the unique breadth of its validation portfolio: defined clinical PK/PD indices enabling rational dosing [44], a characterized host metabolic target [45], a dual macrophage/T-cell immunomodulatory mechanism [46], and proven efficacy in the clinically relevant MG–*E. coli* co-infection model [47]. This multi-layered evidence base positions baicalin as the most translationally advanced phytochemical candidate and establishes a benchmark for the level of characterization that should be aspired to for other natural product candidates.

Quercetin occupies a conceptually pivotal position as the prototype for a pure host-directed therapeutic strategy. The demonstration that robust in vivo efficacy equivalent to tiamulin can be achieved with negligible direct antimicrobial activity (MIC = 1 mg/mL) fundamentally expands the definition of what constitutes an anti-infective agent [40,48]. This work legitimizes the concept that therapeutic outcomes can be attained by enhancing host defense and repair mechanisms rather than by directly attacking the pathogen, with implications extending beyond mycoplasmosis to other chronic mucosal infections.

Luteolin is distinguished by its unique dual-targeting capability, simultaneously engaging a pathogen virulence factor (TatD nuclease) and host inflammatory pathways [49,50]. This polypharmacological profile, which spans direct-acting and host-directed mechanistic categories, positions luteolin as a strategically versatile candidate and establishes virulence factor inhibition as a novel direction for anti-MG drug development. The identification of TatD nuclease as a druggable target in MG represents a particularly significant conceptual advance [50].

The remaining phytochemicals, polydatin [51], andrographolide [52], hydroxytyrosol [53], and methylsulfonylmethane [54], while less extensively characterized, contribute valuable chemical diversity and validate alternative host signaling nodes as therapeutic targets, collectively expanding the repertoire of druggable pathways for MG intervention.

### 3.3. Plant Extracts, Fermentation Products, and the Challenge of Standardization

The evaluation of complex, multi-component preparations reveals a therapeutic category characterized by both significant promise and inherent challenges. Guava leaf (*Psidium guajava* L.) extract stands as the most consistently validated plant extract, with both in vitro and in vivo evidence supporting its antimycoplasmal activity, including MIC values as low as 0.25 µg/mL against field isolates and statistically significant reductions in clinical scores, lesion severity, and pathogen re-isolation rates in an experimental infection model [55,56]. A strategic roadmap for further development through nano-formulation has been proposed to overcome potential bioavailability limitations [55].

Fermented wheat germ extract holds historical significance as the first natural product to demonstrate efficacy comparable to tiamulin, with both treated groups exhibiting markedly reduced lesion scores compared to untreated infected controls and no mycoplasma re-isolated from internal organs of FWGE-treated birds [56]. This pioneering proof-of-concept has shaped the entire field of phytobiotic-based mycoplasmosis management. In contrast, the marginal efficacy of Original XPC (*Saccharomyces cerevisiae* fermentation product), which, despite a numerical reduction in lesion scores, failed to significantly influence the host physiological stress response to MG infection, serves as a critical cautionary demonstration that efficacy is product-specific and cannot be generalized across all fermentation-derived preparations [57,58]. The differential efficacy between fermented wheat germ and yeast products may be attributable to fundamental differences in their bioactive profiles arising from distinct fermentation substrates and processes.

Radix Isatidis Mixtures represent a sophisticated approach to investigating traditional herbal formulations in the clinically relevant context of polymicrobial MG–*E. coli* co-infection, employing a multi-dimensional validation strategy that integrates gene expression, protein interaction, and metabolic pathway analyses [6]. This study is methodologically significant for establishing a robust framework for the mechanistic deconvolution of complex natural product formulations.

### 3.4. Probiotics as a Therapeutic Strategy

Probiotic-based strategies represent a conceptually innovative departure that establishes the host microbiome as a legitimate therapeutic target for controlling a respiratory pathogen. *Bacillus subtilis* (*B. subtilis*) KC1 provides the first complete mechanistic delineation of a gut–lung axis intervention in MG, demonstrating that dietary supplementation can indirectly control pulmonary mycoplasmosis through intestinal microbial community remodeling, restoration of indole metabolism, and systemic AhR-mediated pulmonary protection [45]. *Lactobacillus salivarius* pioneers the concept of respiratory probiotics, demonstrating that direct modulation of the respiratory tract microbiome can simultaneously reduce pathogen burden, correct local immune imbalance, inhibit the JAK/STAT signaling pathway, and restore respiratory microbial community structure [59]. The complementarity of these anatomically and mechanistically distinct strategies, indirect gut–lung axis versus direct respiratory tract localization, suggests that future integrated regimens combining intestinal and respiratory probiotics may provide more comprehensive protection than either approach alone.

### 3.5. Emerging Frontiers: Synthetic Small Molecules and Host RNA Networks

Two emerging frontiers push the boundaries of anti-MG therapeutic strategy in opposite but potentially complementary directions. The narrow-spectrum synthetic inhibitors SM4 and SM9 represent a landmark proof-of-concept for microbiome-sparing antimicrobial therapy. Identified through a rigorous high-throughput screening cascade from a library of 4182 compounds, SM4 and SM9 achieved remarkable in vivo efficacy, reducing airsacculitis by 77.2% and 82.9%, respectively, and decreasing tracheal MG colonization by 0.9 and 2.7 log_10_, without disrupting the richness or evenness of the cecal or tracheal microbiota [60]. This demonstration that effective anti-infective therapy can be decoupled from microbiome perturbation has implications extending well beyond MG to the broader field of antimicrobial development.

Host-encoded non-coding RNA networks represent the most upstream frontier, elucidating the endogenous regulatory circuitry governing the host response to infection. The functional characterization of pro-survival miRNAs, including gga-miR-146c [61] and miR-130b-3p [62]; anti-inflammatory miRNAs, notably the AhR:Arnt/gga-miR-451/YWHAZ axis [63,64]; pro-inflammatory miRNAs such as gga-miR-101-3p [65]; and ceRNA regulatory networks, particularly the Lnc90386/miR-33-5p/JNK1 axis [66], has created a molecular blueprint of the host’s intrinsic defense and pathogenesis programs. While these elements are not themselves therapeutic agents, they constitute a validated landscape of druggable targets for future modalities including miRNA mimics, antagomirs, and lncRNA-targeted silencing strategies.

### 3.6. Nanoemulsion and Liposomal Encapsulation of Phytochemicals for Anti-MG Therapy

The encapsulation of phytochemicals within nanoemulsion or liposomal delivery systems represents a promising strategy to overcome the inherent limitations of crude plant extracts and isolated natural compounds, such as poor aqueous solubility, limited bioavailability, and susceptibility to degradation in the gastrointestinal tract [67]. Liposomal encapsulation employs phospholipid bilayers to form nanoscale vesicles that can entrap hydrophobic bioactive molecules, thereby enhancing their solubility, protecting them from harsh physiological environments, and facilitating sustained release at target sites [68]. This approach has been successfully applied to a range of essential oils, including tea tree oil and *Nigella sativa* oil, with the resulting nanoliposomes demonstrating improved stability, uniform particle size distribution, and enhanced in vitro antibacterial efficacy compared to free compounds [69]. In the context of avian mycoplasmosis, where chronic respiratory infection necessitates prolonged therapeutic exposure, such delivery platforms offer the additional advantage of potentially reducing the required dose and dosing frequency while maintaining therapeutic concentrations at the respiratory mucosa.

Although direct evidence for nanoencapsulated phytochemicals against MG remains limited, recent studies in related poultry pathogens provide a strong preclinical rationale for this approach. Ahmad and colleague demonstrated that nanoliposomal *Nigella sativa* oil co-formulated with the immunostimulatory adjuvant monophosphoryl lipid A (NS-MPLA) conferred significant protection against *Salmonella* pullorum in broiler chicks, promoting bacterial clearance, upregulating mucosal immune markers (IgA, MUC2), and stimulating pro-inflammatory cytokine expression [70]. Similarly, tea tree oil nanoliposomes (TTONL) prepared via thin-film hydration exhibited dose-dependent bactericidal activity against avian pathogenic *E*. *coli* and alleviated intestinal pathology in experimentally challenged chickens, partly through suppression of NLRP3 and NF-κB (p65) mRNA expression [69]. These findings are mechanistically relevant to MG control, given that many phytochemicals reviewed in the present work, including baicalin, luteolin, and quercetin, similarly exert their effects via host immunomodulation and suppression of NF-κB-driven inflammation [71]. The convergence of nanocarrier technology with phytochemical pharmacology therefore holds considerable translational potential. Future studies should focus on optimizing encapsulation parameters (e.g., lipid composition, surface charge, particle size) for pulmonary delivery via aerosol or oral administration, evaluating pharmacokinetic profiles in poultry, and directly assessing nanoencapsulated anti-MG candidates in vivo.

### 3.7. Strategic Synthesis and Future Directions

A synthesis of the anti-MG therapeutic landscape permits several strategic conclusions. First, the field has successfully validated multiple non-antibiotic modalities that can achieve clinically meaningful efficacy while offering advantages in resistance resilience, microbiome preservation, or both. The demonstration that fermented wheat germ extract [36], quercetin [39], and the narrow-spectrum inhibitors SM4 and SM9 [60] can achieve outcomes comparable or equivalent to conventional antibiotics provides robust evidence that effective MG control need not depend solely on traditional chemotherapy. Second, the most promising therapeutic candidates are characterized by multi-target or host-directed mechanisms that address the immunopathological component of MG disease rather than merely eliminating the pathogen. Third, the molecular convergence of diverse agents, phytochemicals, probiotics, and miRNAs, on a limited set of host signaling pathways suggests that rational combinations of agents targeting distinct nodes within these networks may achieve synergistic effects. The path forward for the field likely lies in the strategic integration of these diverse modalities into combination regimens that leverage their complementary strengths: the potency and defined dosing of novel pleuromutilin derivatives; the resistance resilience and microbiome preservation of narrow-spectrum inhibitors and host-directed phytochemicals; the ecological restoration capacity of probiotics; and the unprecedented specificity of future miRNA-based therapeutics. The collective body of work reviewed herein represents not merely an accumulation of individual therapeutic candidates but the emergence of a mature, mechanistically sophisticated, and therapeutically diversified field poised for translational advancement.

## 4. Mechanistic Taxonomy of Anti-MG Action

The mechanisms of action can be systematized into two overarching strategies: direct pathogen-directed effects and indirect host-directed effects. A significant number of natural compounds operate through a confluence of both [50].

### 4.1. Direct Pathogen-Directed Mechanisms

The direct targeting of MG viability, structural integrity, and key virulence factors represents the most intuitive and historically established paradigm in anti-mycoplasmal chemotherapy [45]. Unlike host-directed strategies that seek to modulate the host’s immune response to create an environment refractory to infection, these mechanisms aim to neutralize the pathogen through direct physicochemical or biochemical intervention. The agents employing such mechanisms span a remarkable breadth of chemical complexity, from crude multi-component plant extracts to precisely designed synthetic small molecules and semi-synthetic antibiotic derivatives [72]. A systematic analysis of these direct-acting mechanisms reveals a diverse mechanistic arsenal that can be organized along a continuum from broad, multi-target disruption to exquisitely specific molecular inhibition [41]. This section critically examines the five principal categories of direct pathogen-directed mechanisms, evaluating their molecular bases, their representation across different agent classes, and their therapeutic implications.

#### 4.1.1. Disruption of Cell Membrane Integrity and Conformation

This mechanism constitutes the most fundamental and phylogenetically ancient antimicrobial strategy, operating through the physical compromise of the pathogen’s sole lipid bilayer. It is particularly salient for *Mycoplasma* species, which, as wall-less bacteria, rely entirely on their cell membrane for structural integrity, selective permeability, and interaction with the host environment. The multi-phytochemical composition of guava (*Psidium guajava*) leaf extract, encompassing flavonoids, tannins, saponins, and phenolic compounds, is proposed to exert its antimycoplasmal effects through complementary membrane-disruptive pathways, including direct perturbation of lipid bilayer architecture, chelation of divalent metal ions essential for membrane stabilization, and interference with membrane-associated enzymatic processes [73,74]. This polypharmacological assault on membrane integrity exemplifies the multi-target mode of action characteristic of many plant-derived antimicrobials, a property that may reduce the probability of resistance emergence compared to agents acting on a single molecular target. At the opposite end of the chemical complexity spectrum, the narrow-spectrum synthetic inhibitors SM4 and SM9 achieve their antimycoplasmal effect through a more specific, albeit still membrane-targeting, mechanism: the induction of significant alterations in MG cell membrane conformation [60]. While the precise biophysical nature of this conformational change remains to be fully elucidated, the observation that these compounds selectively affect MG while sparing commensal bacteria suggests that they may exploit unique structural features of the mycoplasmal membrane, such as its distinctive sterol composition or the specific organization of its membrane protein complexes. Resistance may emerge against any inhibitory substance, whether derived from natural sources or produced synthetically, through the selection of resistant mutants, and such acquired resistance is heritable. Nevertheless, membrane-targeting strategies may offer a pharmacokinetic/pharmacodynamic advantage by acting on a supramolecular structure whose fundamental biophysical properties are less readily altered by single-point mutations than classical protein or nucleic acid targets, thereby potentially raising the barrier to resistance development.

#### 4.1.2. Inhibition of Pathogen Adhesion and Colonization

Natural products frequently employ an anti-virulence strategy that, rather than killing the pathogen outright, disarms it of its ability to establish and maintain infection. Cytoadherence to the respiratory epithelium is an essential early step in MG pathogenesis, mediated primarily by a specialized polar tip organelle and a suite of adhesin proteins, among which pMGA1.2 is a well-characterized and critical mediator. A notable convergence has emerged across mechanistically and chemically distinct anti-MG agents on the suppression of pMGA1.2 expression. The diterpenoid lactone andrographolide, derived from *Andrographis paniculata*, significantly downregulates pMGA1.2 expression as part of its integrated anti-inflammatory and anti-apoptotic profile [52]. Similarly, the flavonoid luteolin suppresses the expression of this adhesin both in vivo and in vitro, thereby inhibiting pathogen colonization while simultaneously restoring host immune competence [49]. Remarkably, this anti-adhesin mechanism extends beyond exogenous small molecules to host-encoded regulatory RNAs: the protective miRNA miR-33-5p, when freed from its molecular sponge Lnc90386, inhibits pMGA1.2 expression as part of its broader anti-inflammatory and anti-apoptotic function [66]. This convergence of phytochemicals and host miRNAs on a single virulence determinant is striking and suggests that pMGA1.2 expression is controlled by signaling pathways, potentially including JAK/PI3K/AKT, IL-17/NF-κB, and JNK, that are susceptible to both pharmacological and endogenous modulation. The anti-adhesion approach offers the strategic advantage of attenuating pathogenicity without imposing the strong selective pressure for resistance that accompanies bactericidal mechanisms, and the growing number of agents capable of suppressing pMGA1.2 expression positions this adhesin as a validated and therapeutically tractable anti-virulence target [10].

#### 4.1.3. Inhibition of Virulence Factor Activity

This mechanism represents the most molecularly precise of the direct-acting strategies, targeting a specific pathogenic effector rather than a structural component or biosynthetic process. The identification and validation of the TatD nuclease as a druggable virulence target in MG by Liu and colleagues constitutes a seminal advance in this domain [40]. TatD is a key MG virulence factor that localizes to the mycoplasmal cytoplasm and is released into the host environment via extracellular vesicles. Once internalized by host cells, TatD retains its enzymatic nuclease activity and induces pyroptosis, a highly inflammatory form of programmed cell death, through a mechanism associated with the cGAS-STING pathway. Through a combination of network pharmacology, molecular docking, and biomembrane layer interference experiments, luteolin was identified as a direct inhibitor of the TatD nuclease, physically binding to the enzyme and suppressing its catalytic activity. Importantly, luteolin not only inhibited the enzymatic function of TatD but also mitigated the cellular damage caused by this virulence factor. This dual action, direct enzymatic inhibition and attenuation of downstream cytotoxicity, positions TatD as a validated therapeutic target and luteolin as a prototype virulence factor inhibitor. By establishing that nucleases can serve as druggable targets in *Mycoplasma* species, this work opens a novel direction for rational anti-MG drug development centered on the selective neutralization of specific pathogenic effector molecules. The virulence factor inhibition strategy offers the theoretical advantages of extreme target specificity, minimal impact on commensal microbiota, and the preservation of the host’s endogenous microbial ecology.

#### 4.1.4. Inhibition of Macromolecular Synthesis

Natural products are also capable of inhibiting bacterial protein synthesis through ribosomal targeting, which constitutes the classical mechanism of action for the most well-established classes of antimycoplasmal antibiotics: the pleuromutilins and the macrolides. Both classes exert their bacteriostatic or bactericidal effects by binding to the 50S subunit of the bacterial ribosome, albeit at distinct but partially overlapping binding sites within the peptidyl transferase center. The first-generation pleuromutilin tiamulin serves as the benchmark positive control in numerous studies evaluating natural alternatives, consistently demonstrating robust in vivo efficacy against MG [38,39]. The third-generation macrolide Tylvalson (ECO Animal Health) provides enhanced potency and an expanded spectrum, and its therapeutic potential can be further augmented through combination with zinc oxide nanoparticles, which simultaneously enhance efficacy and reduce tissue antibiotic residues [20]. The most significant recent advances in this mechanistic class, however, are the novel semi-synthetic pleuromutilin derivatives amphenmulin and p-furoylamphenmulin, which exhibit exceptionally low minimum inhibitory concentrations against MG, 0.0039 μg/mL and 0.00195 μg/mL, respectively, and well-characterized concentration-dependent bactericidal activity [40,41]. The comprehensive PK/PD integration performed for these derivatives has identified the AUC24h/MIC ratio as the PK/PD index best correlating with antimicrobial effect and has generated the quantitative therapeutic targets necessary for rational clinical dosing regimen design. While these novel derivatives represent a refinement rather than a departure from the established ribosomal inhibition mechanism, their dramatically enhanced potency and optimized pharmacokinetic profiles demonstrate the significant gains that can be achieved through iterative medicinal chemistry optimization of validated pharmacophores. A limitation inherent to this mechanism, however, is the documented capacity of mycoplasmas to develop resistance to pleuromutilins and macrolides through mutations in the 23S rRNA gene and the acquisition of efflux determinants, underscoring the importance of antimicrobial stewardship and the potential value of combination therapy with agents employing alternative mechanisms.

#### 4.1.5. Multi-Target Metabolic Interference in Pathogens

This mechanism represents a sophisticated strategy that exploits pathogen-specific metabolic vulnerabilities induced by the infection process itself. Chen and colleagues elucidated a novel metabolic axis in MG pathogenesis, demonstrating that MG infection induces the accumulation of ceramide, a bioactive sphingolipid, via the de novo synthesis pathway, and that this ceramide accumulation is critical for MG proliferation [66]. The mechanistic dissection of this pathway revealed a sequential cascade initiated by MG-induced upregulation of stromal interaction molecule 1 (STIM1) expression, leading to Calcium overload, which in turn drives oxidative stress and endoplasmic reticulum (ER) stress, culminating in ceramide accumulation. Pharmacological inhibition of this de novo ceramide synthesis pathway significantly suppressed both MG proliferation and the associated inflammatory injury, validating the pathway as a therapeutic target. In this context, baicalin was identified as an agent capable of interrupting this pathogenic cascade at its initiating step by downregulating STIM1 expression, thereby restoring Calcium homeostasis, mitigating oxidative stress, and alleviating ER stress and ceramide accumulation. This mechanism is conceptually distinct from the other direct-acting mechanisms reviewed: rather than targeting a constitutive structural feature, a virulence factor, or a core biosynthetic process of the pathogen, baicalin targets a host metabolic pathway that MG has hijacked to create a favorable intracellular environment for its proliferation. This strategy of targeting pathogen-induced host metabolic vulnerabilities occupies an intriguing mechanistic interface between direct antimicrobial action and host-directed therapy, as it targets a host factor (STIM1) but does so to disrupt a metabolic condition (ceramide accumulation) upon which the pathogen depends. This “metabolic trap” approach may offer the advantage of targeting a pathway that is essential for pathogen proliferation but not for normal host cellular function, potentially achieving efficacy with reduced host toxicity.

In summary, a comparative assessment of these five direct pathogen-directed mechanisms reveals a mechanistic landscape of remarkable diversity, spanning from the broad, multi-target membrane disruption of crude plant extracts to the exquisitely specific inhibition of a single virulence factor enzyme. Several integrative themes emerge from this analysis. First, there exists a clear mechanistic gradient in target specificity, with membrane disruption and ribosomal inhibition at the broad-spectrum end, and virulence factor inhibition and host metabolic interference at the narrow-spectrum, pathogen-specific end. This gradient has direct implications for resistance emergence risk, commensal microbiota preservation, and therapeutic index, with the more specific mechanisms generally offering advantages in these parameters. Second, certain agents, most notably luteolin, exemplify a dual-targeting strategy that spans multiple mechanistic categories. Luteolin simultaneously inhibits the TatD virulence factor through direct binding [40], suppresses pMGA1.2-mediated adhesion [52], and modulates host MAPK signaling [54], a convergence of virulence factor inhibition, anti-adhesion, and host-directed mechanisms within a single molecule that defines an emerging paradigm of anti-infective polypharmacology. Third, the field is witnessing a strategic shift from purely empirical antimicrobial screening toward mechanism-informed drug design, as exemplified by the PK/PK-optimized pleuromutilin derivatives [36] and the pathway-targeted development of baicalin for STIM1/ceramide axis intervention [66]. Fourth, the anti-adhesion mechanism, represented by the convergent activity of andrographolide [54], luteolin [52], and miR-33-5p on pMGA1.2 [60], emerges as a particularly promising and therapeutically underexploited strategy that merits further investigation, including the structural characterization of the regulatory pathways governing adhesin expression and high-throughput screening for selective pMGA1.2 downregulators. Collectively, the diversity and sophistication of the direct pathogen-directed mechanisms reviewed herein provide a rich and expanding repertoire of therapeutic options. While vaccination and mycoplasma eradication programs remain the cornerstone strategies for achieving Mycoplasma-free flocks and reducing antibiotic reliance, their practical limitations, including the high cost and stringent biosecurity measures required for sustained mycoplasma freedom, underscore the urgent need for complementary therapeutic approaches. The strategic integration of these direct pathogen-directed mechanisms with the host-directed approaches reviewed below therefore holds considerable promise for the development of next-generation, resistance-resilient anti-MG therapeutic regimens that can serve as an adjunct to, rather than a replacement for, established preventive measures.

### 4.2. Host-Directed Mechanisms

An important but often underappreciated aspect of MG pathogenesis is the central role of immune evasion in driving both chronic infection and the associated production losses. Unlike acute bacterial pathogens that rely on rapid replication and toxin-mediated tissue destruction, MG employs a sophisticated repertoire of immune evasion strategies to establish persistent colonization within the host respiratory tract [39]. Central to this immune evasion is the high-frequency antigenic variation in surface-exposed adhesins, most notably the pMGA family of lipoproteins, which enables MG to continuously evade antibody-mediated clearance while maintaining essential cytoadhesion functions [74]. Beyond antigenic variation, MG can invade and survive within non-phagocytic host cells, thereby shielding itself from both humoral and cellular immune effectors [75]. Furthermore, MG actively manipulates host immune signaling, including the dysregulation of microRNAs and the suppression of key inflammatory mediators, creating a localized state of immunosuppression that facilitates persistence [75].

The biological cost of this prolonged host, pathogen standoff is substantial. The host immune system mounts a sustained inflammatory response in an attempt to eliminate the pathogen, but the continuous antigenic switching and intracellular sequestration mean that MG is effectively a constantly moving target, hence the concept of the “biological cost of chasing MG.” This unresolved inflammation drives progressive tissue damage in the respiratory tract, spleen, and thymus, ultimately manifesting as the production losses characteristic of chronic MG infection: reduced body weight gain, impaired feed conversion, depressed egg production, and increased susceptibility to secondary infections [39,73]. Critically, this immune-mediated pathology often exceeds the damage attributable to direct pathogen cytolysis, positioning host immune dysregulation, rather than pathogen burden perse, as the primary driver of economic losses [39].

It is precisely this recognition, that a substantial component of MG pathology is attributable not to direct pathogen-mediated cytolysis but to a dysregulated and excessive host inflammatory response, that has driven the emergence of host-directed therapeutic strategies as a fundamental paradigm shift in the approach to controlling MG infection. Rather than targeting the pathogen directly, a strategy that inevitably imposes selective pressure for resistance, these interventions seek to modulate the host’s own immune and cellular responses, repair infection-induced tissue damage, and strengthen intrinsic defense barriers to create a local environment that is inhospitable to pathogen persistence and propagation. This approach is particularly well-suited to chronic MG infection, as it directly addresses the immune evasion–chronic inflammation–production loss axis described above. The agents employing host-directed mechanisms are predominantly natural product-derived phytochemicals, probiotics, and host-encoded regulatory RNAs, and their mechanisms collectively span a continuum from the suppression of specific pro-inflammatory signaling cascades to the restoration of broad immune homeostatic balance and physical barrier integrity. This section provides a systematic analysis of the four principal categories of host-directed mechanisms, evaluating their molecular targets, functional outcomes, and therapeutic implications.

#### 4.2.1. Modulation of Host Signaling Pathways to Suppress Inflammation

The nuclear factor-κB (NF-κB) transcription factor family emerges as the central signaling node targeted by the majority of these agents, reflecting its master regulatory role in orchestrating the expression of pro-inflammatory cytokines, chemokines, and anti-apoptotic factors. The NF-κB-centric mechanisms can be further stratified by the upstream receptors and adaptor proteins through which the pathway is engaged. At the level of Toll-like receptor (TLR)-initiated signaling, polydatin selectively inhibits the TLR6/MyD88/NF-κB axis, restraining NF-κB-p65 nuclear translocation and thereby suppressing the transcriptional program driving IL-6, IL-1β, and TNF-α expression [54]. Quercetin, operating through a distinct TLR specificity, suppresses the TLR2/MyD88/NF-κB pathway while simultaneously attenuating MG-induced oxidative stress, establishing a mechanistic profile that integrates antioxidant and anti-inflammatory activities [52]. At the level of interleukin-17-initiated signaling, both luteolin and baicalin converge on the IL-17/NF-κB axis, though in distinct infection contexts: luteolin suppresses this pathway in MG mono-infection, downregulating IL-17A, TRAF6, phosphorylated p65, and phosphorylated IκBα [49], while baicalin targets the same pathway in the clinically relevant MG–*E. coli* co-infection model, additionally suppressing downstream effectors including CXCL1, CXCL2, MMP1, and MUC5AC [47]. A third NF-κB-centric mechanism targets the inflammasome arm of innate immunity: hydroxytyrosol blocks the NF-κB/NLRP3/IL-1β cascade, effecting a broad downregulation of NLRP3, caspase-1, and multiple pro-inflammatory cytokines, thereby providing dual anti-inflammatory and anti-apoptotic protection [53]. A recent study demonstrated that *Scutellaria baicalensis*-derived exosome-like nanoparticles (SBELNs), when administered via aerosol inhalation, exhibit efficient lung tropism, a biodistribution pattern that is dependent on the route of administration rather than an intrinsic tissue-homing property, and alleviate MG-induced inflammatory lung injury by delivering miR159a to target CNGA1, thereby restoring intracellular Calcium homeostasis, preserving mitochondrial function, and suppressing NF-κB-driven inflammation [75]. While the aerosol route enables direct pulmonary delivery and minimizes systemic exposure, it also raises considerations regarding operator safety during large-scale poultry application, an aspect that warrants further evaluation before field deployment.

The mitogen-activated protein kinase (MAPK) pathways constitute a second major signaling module targeted by host-directed agents, often in conjunction with NF-κB inhibition. Andrographolide inhibits the MG-induced JAK/PI3K/AKT signaling pathway, an intervention that simultaneously suppresses inflammation and apoptosis while downregulating the expression of the MG adhesin protein pMGA1.2 [52]. The integration of anti-inflammatory, anti-apoptotic, and anti-adhesive effects through the inhibition of a single upstream kinase cascade exemplifies an efficient polypharmacology. Methylsulfonylmethane (MSM) adopts a dual-pathway inhibitory strategy, simultaneously suppressing both the NF-κB and ERK/JNK-MAPK signaling modules in tracheal tissue and macrophages, providing broad-spectrum inflammatory suppression through a chemically simple, dietary-derived scaffold [54]. Baicalin, whose mechanisms span both direct antimicrobial and host-directed categories, suppresses the TLR4-p38 MAPK/NF-κB pathway specifically in the context of restoring macrophage polarization balance, demonstrating how the same signaling pathway can be targeted by the same agent to achieve distinct functional outcomes depending on the cellular context [46]. The frequent pharmacological activity of diverse phytochemicals against the NF-κB and MAPK signaling modules strongly suggests that these pathways represent the principal druggable nodes through which MG-induced inflammation can be pharmacologically attenuated.

#### 4.2.2. Restoration of Immune Homeostasis and Barrier Function

Beyond the suppression of individual signaling pathways, addressing the broader immunological and structural consequences of MG infection constitutes a second major category of host-directed mechanisms. MG infection characteristically disrupts the delicate balance between pro-inflammatory and anti-inflammatory macrophage populations (M1/M2) and T-helper cell subsets (Th1/Th2), creating an immunological environment that perpetuates chronic inflammation and tissue damage. Baicalin has been shown to rectify both imbalances simultaneously: it disrupts MG-induced M1 macrophage activation, restores the M1/M2 ratio, and rebalances Th1/Th2 differentiation in the lungs of infected chickens [46]. Ultrastructural analyses confirmed that these immunological corrections were accompanied by the restoration of mitochondrial morphology, reduction in cytoplasmic vacuolation, and normalization of chromatin architecture, indicating that the immunomodulatory effects translate into tangible cellular and tissue-level repair. Quercetin employs a mechanistically distinct but functionally convergent approach to restoring immune homeostasis. Rather than merely suppressing M1 activation, quercetin actively promotes the polarization of macrophages toward the reparative M2 phenotype through the induction of fatty acid oxidation (FAO) and activation of the PI3K/AKT signaling pathway, thereby skewing the immune response toward a Th2 phenotype that favors tissue repair and resolution of inflammation [48]. This active promotion of a reparative phenotype, as opposed to simple suppression of a pro-inflammatory one, represents a more sophisticated immunomodulatory strategy.

Complementing these immunological corrections, quercetin has been further demonstrated to directly restore the physical integrity of the respiratory mucosal barrier, a critical host defense structure that MG infection compromises. Quercetin achieves this restoration through a dual mechanism. First, it upregulates tight junction proteins, thereby sealing the paracellular spaces between epithelial cells. While MG can invade host cells directly intact tight junctions serve to maintain overall epithelial barrier integrity, limit secondary bacterial translocation, and prevent the leakage of luminal pro-inflammatory stimuli that would otherwise exacerbate MG-driven immunopathology [39]. Second, quercetin promotes the expression of secretory immunoglobulin A (sIgA), the principal adaptive immune effector at mucosal surfaces, which contributes to pathogen neutralization and immune exclusion at the apical epithelial interface [39]. This concerted restoration of both innate barrier function and adaptive mucosal immunity provides a comprehensive repair mechanism. The probiotic *Lactobacillus salivarius* contributes an additional dimension to immune homeostatic restoration by rectifying MG-induced Th1/Th2 imbalance through the suppression of the JAK/STAT signaling pathway in the tracheal mucosa [56]. Notably, the Th2-skewing, barrier-restoring phenotype induced by quercetin and L. *salivarius* mirrors that achieved by the probiotic *B. subtilis* KC1 through its distinct gut–lung axis mechanism, suggesting that the promotion of a Th2-dominant, tissue-reparative immune milieu may represent a convergent therapeutic endpoint achievable through pharmacologically and microbiologically diverse interventions.

#### 4.2.3. Indirect Microbiome-Mediated Immunomodulation via the Gut–Lung Axis

This category represents a mechanistically unique and conceptually innovative host-directed strategy. Unlike the phytochemicals that directly engage host signaling pathways at the site of infection, *B. subtilis* KC1 operates entirely through the remote modulation of the intestinal microbial ecosystem [45]. The mechanistic chain is strikingly sequential, and the results showed that dietary *B. subtilis* KC1 supplementation selectively enriched the beneficial intestinal bacterium *Bifidobacterium animalis*; this compositional shift restores the indole metabolic dysfunction caused by MG infection; the elevated systemic indole levels subsequently enhance the activation of the aryl hydrocarbon receptor (AhR) in the pulmonary compartment; and AhR activation, in turn, improves epithelial barrier function and suppresses lung inflammation. This mechanism establishes that the gut microbiota represents a manipulable node within the gut–lung axis for controlling respiratory MG infection, a concept that had not been previously applied to this pathogen. The identification of indole as the key effector metabolite is mechanistically informative, as indole is a well-characterized AhR ligand with documented roles in promoting mucosal barrier integrity and regulating inflammatory responses. The AhR activation achieved indirectly by *B. subtilis* KC1 through indole restoration converges with the AhR activation that drives the transcription of the protective, anti-inflammatory miRNA gga-miR-451, suggesting that the gut–lung axis and host miRNA defense networks may be functionally integrated through shared receptor systems. Notably, the host-directed nature of this strategy is to modulate endogenous anti-inflammatory and barrier-enhancing pathways rather than directly targeting the pathogen, and confers a potential advantage in cross-species applicability. Because AhR-mediated epithelial barrier enhancement and inflammation suppression are broadly protective host responses rather than MG-specific interventions, this mechanism may, in principle, confer resilience against other avian mycoplasmas, such as Mycoplasma synoviae, that similarly exploit mucosal barrier disruption and excessive inflammation for pathogenesis. However, this theoretical broad-spectrum benefit requires empirical validation, as the specific contributions of gut–lung axis signaling to the control of other avian mycoplasma diseases remain unexplored. Moreover, the species-specificity of the microbial- metabolite-host receptor cascade merits careful investigation before generalization beyond MG, in particular, whether indole-mediated AhR activation is equally effective across different Mycoplasma species that may employ distinct immune evasion strategies.

#### 4.2.4. Modulation of Host Non-Coding RNA Defense Networks

The modulation of host non-coding RNA defense networks represents the most recently elucidated and mechanistically sophisticated category of host-directed intervention. These strategies, which target host miRNAs and lncRNAs rather than exogenous pharmacophores, represent potential future therapeutic modalities based on miRNA mimics, antagomirs, or siRNA-mediated lncRNA knockdown. The pro-defense miRNAs are host-protective factors whose therapeutic augmentation could reinforce intrinsic cellular resilience. gga-miR-146c, upregulated during MG infection, activates the TLR6/MyD88/NF-κB pathway through targeting MMP16, simultaneously promoting cell survival and proliferation while enhancing innate immune signaling [45]. miR-130b-3p operates through a complementary mechanism, activating the PI3K/AKT/NF-κB axis through repression of its target PTEN to counteract MG-inhibited cell proliferation [66]. Both miRNAs converge on NF-κB activation as a pro-survival signal, contrasting with the NF-κB-suppressive mechanisms of the phytochemicals and highlighting the context-dependent duality of this transcription factor, which can mediate both protective and pathological outcomes depending on the magnitude, duration, and cellular context of its activation.

**Figure 1:**
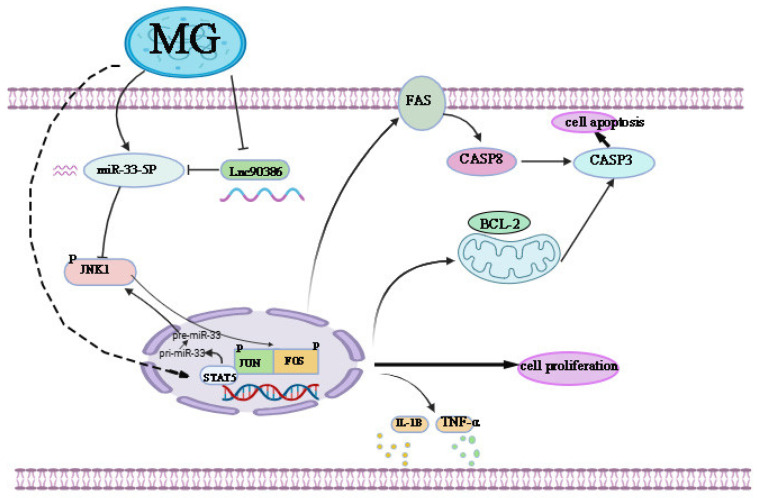
Illustration of the potential roles of Lnc90386/miR-33-5p in DF-1 cells during MG infection. Reproduced from the work of Sun et al. (2022) [66], distributed under the terms of the Creative Commons Attribution License (CC BY). “⊣” represents inhibition, and “→” represents activation.

The therapeutic targeting of ceRNA networks offers an alternative strategy that exploits the endogenous regulatory architecture of the host cell. The Lnc90386/miR-33-5p/JNK1 axis exemplifies this approach. miR-33-5p is a protective miRNA that suppresses JNK1-mediated inflammation and apoptosis, and it also inhibits the expression of the MG adhesin pMGA1.2. However, its protective function is constitutively restrained by Lnc90386, which acts as a molecular sponge that sequesters miR-33-5p and prevents it from engaging its JNK1 target (Figure 1) [66]. Therapeutic knockdown of Lnc90386, therefore, represents a host-targeted strategy that removes this molecular brake, releasing endogenous miR-33-5p to exert its full protective anti-inflammatory, anti-apoptotic, and anti-adhesive effects. This strategy offers the conceptual advantage of harnessing an endogenous, physiologically regulated defense mechanism rather than introducing an exogenous pharmacophore, potentially reducing off-target effects.

In summary, a comparative analysis of the host-directed mechanisms reveals several overarching themes. First, there is a striking hierarchical organization of host-directed interventions, ranging from the targeting of individual signaling molecules within specific pathways, through the restoration of multicellular immune homeostatic balances, to the modulation of entire microbial ecosystems and host non-coding RNA regulatory networks. This hierarchy corresponds to an increasing level of biological complexity and, potentially, to increasingly physiological and sustainable therapeutic strategies. Second, the NF-κB pathway emerges as the dominant signaling node for pharmacological intervention, targeted by the majority of phytochemicals through distinct upstream receptors and adaptors. The fact that NF-κB can be both suppressed (by anti-inflammatory agents) and activated (by pro-survival miRNAs) to achieve protective outcomes underscores the critical importance of context, magnitude, and duration in determining the net effect of NF-κB pathway modulation. Third, signaling pathway convergence is a pervasive feature of this field: the JAK/STAT pathway is targeted by andrographolide and L. *salivarius*; the AhR pathway is activated by *B. subtilis* KC1 and drives gga-miR-451 transcription; the MAPK cascades are modulated by MSM and baicalin. This convergence suggests that a finite set of master regulatory pathways orchestrates the host response to MG and that multi-agent combinations targeting distinct nodes within these same pathways may achieve synergistic effects. Fourth, the complementarity between exogenous phytochemicals and endogenous regulatory networks is a particularly promising area for future investigation. The demonstration that probiotic-derived metabolites can activate the same AhR complex that drives protective miRNA expression, and that phytochemicals can modulate the same pathways regulated by host miRNAs, suggests the potential for integrated therapeutic regimens that combine probiotics, phytochemicals, and miRNA-based modalities. Collectively, the host-directed mechanisms reviewed herein have fundamentally expanded the therapeutic landscape for MG infection, offering strategies that address the underlying immunological dysfunction driving pathology rather than merely eliminating the microbial trigger, and thereby holding promise for more durable and resistance-resilient therapeutic outcomes.

## 5. Challenges and Future Directions

### 5.1. Critical Knowledge Gaps

Despite the encouraging data reviewed herein, the current evidence base for natural anti-MG products remains nascent, and substantial knowledge gaps must be addressed before these agents can be recommended for routine field application. Firstly, scarcity of in vivo data. The majority of available studies are limited to in vitro determination of MIC values; rigorous in vivo efficacy trials conducted under conditions that simulate commercial production environments are comparatively rare. Notable exceptions include the Toldin CRD study by Hashem and colleagues [76], the TCM investigation by Wang and colleagues (2025) [77], and the guava extract evaluation by Shakal and colleagues (2023) [55], but these studies vary in design, challenge model, and outcome measures, complicating cross-study comparisons. Secondly, limited mechanistic understanding. Although the broad mechanisms of natural products have been delineated (membrane disruption, oxidative stress, metal chelation), the specific molecular targets within MG remain poorly characterized. For example, the precise binding sites of phycocyanin or essential oil constituents on mycoplasmal structures have not been elucidated. Detailed mechanistic studies are essential for rational product development and the anticipation of potential resistance emergence. Thirdly, standardization challenges. Natural products are inherently variable in composition, reflecting differences in plant cultivar, geographic origin, harvest time, extraction method, and storage conditions. The establishment of standardized extraction protocols, chemical fingerprinting, and quality control parameters is essential for ensuring batch-to-batch consistency and reliable therapeutic outcomes.

### 5.2. Formulation and Delivery

The bioavailability of natural bioactive compounds presents a significant challenge, particularly for hydrophobic constituents such as curcumin and certain essential oil components. Nano-formulation strategies—exemplified by the study of curcumin NPs—offer a promising approach to enhancing aqueous dispersibility, improving tissue penetration, and prolonging residence time at target sites. The recommendation by Shakal and colleagues to evaluate guava leaf extract as a nano-preparation reflects growing recognition that advanced drug delivery systems may be critical for translating in vitro activity into in vivo efficacy [21].

### 5.3. Regulatory and Practical Considerations

The pathway to commercial adoption of natural anti-MG products involves navigating a complex regulatory landscape. In many jurisdictions, natural products intended for therapeutic use in food-producing animals must satisfy requirements for safety, efficacy, and residue depletion that are comparable to those applied to conventional veterinary pharmaceuticals. Harmonization of regulatory standards, development of appropriate monographs, and engagement with industry stakeholders will be necessary to facilitate market access [78].

### 5.4. Research Priorities

Based on this analysis, the following research priorities are identified: (1) rigorous in vivo efficacy trials using standardized challenge models, clinically relevant outcome measures (clinical scores, lesion scores, microbiological clearance, growth performance), and appropriate comparator antibiotics; (2) elucidation of molecular mechanisms underlying both direct antimycoplasmal activity and host-directed immunomodulation, employing techniques such as transcriptomics, proteomics, and advanced imaging; (3) development of standardized extraction and formulation protocols, including chemical characterization using chromatographic and spectroscopic methods; (4) investigation of nano-formulation and targeted delivery systems to enhance bioavailability and therapeutic index; (5) pharmacoeconomic analyses comparing the cost effectiveness of natural products with conventional antibiotic regimens under different production scenarios; and (6) surveillance for potential resistance emergence during prolonged natural product use, including assessment of minimum inhibitory concentration drift and characterization of any adaptive mutations.

## 6. Conclusions

The growing prevalence of antimicrobial resistance among *Mycoplasma gallisepticum* field isolates represents a formidable challenge to the sustainability of conventional antibiotic-based control programs. Natural products derived from medicinal plants, essential oils, and microalgae offer a compelling alternative paradigm, distinguished by multi-target mechanisms that circumvent existing resistance pathways and confer ancillary benefits encompassing immunomodulation, anti-inflammatory protection, and growth promotion.

The evidence reviewed in this article demonstrates that several natural products, including *Psidium guajava* leaf extract, traditional Chinese medicine formulations, curcumin nanoparticles, essential oil mixtures such as Toldin CRD, and *Spirulina platensis*, possess measurable in vitro and, in some cases, validated in vivo antimycoplasmal activity. The absence of cross-resistance correlations with macrolides and other conventional antibiotics, particularly well-documented for *Spirulina*, provides a strong scientific rationale for their investigation as resistance-bypassing therapeutics.

However, the translation of these promising findings into practical, field-ready interventions requires substantial additional investment in rigorous in vivo validation, mechanistic elucidation, standardization, and formulation science. The ultimate goal is not the wholesale replacement of antibiotics, which will retain a vital role in managing severe outbreaks, but the development of integrated strategies that deploy natural products as first-line prophylactic and therapeutic agents, reserving conventional antibiotics for situations where they are most critically needed. Such an approach aligns with the principles of antimicrobial stewardship and the One Health framework, contributing to the preservation of antibiotic efficacy for future generations while supporting the productivity and profitability of the global poultry industry.

## Figures and Tables

**Table 1:** Mutations in MG associated with antimicrobial resistance.

Gene Target	Mutation Site (Nucleotide)	Amino Acid Change	Antibiotic Class Affected	Specific Drugs	Resistance Mechanism & Notes
*23S rRNA*	A2058G	N/A* (rRNA)	Macrolides	Tylosin, Tilmicosin	Target alteration in domain V of the peptidyl transferase center. This is a primary determinant of high-level macrolide resistance in field isolates [28].
*23S rRNA*	A2059G	N/A* (rRNA)	Macrolides	Tylosin, Tilmicosin	Target alteration in domain V. Confers cross-resistance to 16-membered macrolides [28].
*23S rRNA*	A2503T	N/A* (rRNA)	Pleuromutilins, Phenicols, Lincosamides	Tiamulin, Valnemulin, Florfenicol, Lincomycin	Target alteration. This mutation confers resistance to pleuromutilins and is associated with decreased susceptibility to chloramphenicol and florfenicol [29].
GyrA	C241T (*E. coli* numbering equivalent)	Ser81Gly	Fluoroquinolones	Enrofloxacin, other fluoroquinolones	Alteration in the Quinolone Resistance-Determining Region (QRDR) in DNA gyrase subunit A. A critical mutation for fluoroquinolone resistance [25].
GyrA	G249T (*E. coli* numbering equivalent)	Ser83Ile	Fluoroquinolones	Enrofloxacin, other fluoroquinolones	Another key mutation in the QRDR of GyrA, found in resistant clinical isolates [25].
ParC	C239T (*E. coli* numbering equivalent)	Ser80Leu	Fluoroquinolones	Enrofloxacin, other fluoroquinolones	Alteration in the QRDR in topoisomerase IV subunit A. Resistance typically requires mutations in both GyrA and ParC [25,30].
*rpoC*	305,216 (Genomic position)	High-impact mutation	Not specified	Not specified	This mutation in the RNA polymerase beta’ subunit gene is predicted to have a high impact on transcription and is associated with antibiotic resistance in a genomic analysis [31].
*dxr*	109,331 (Genomic position)	High-impact mutation	Not specified	Not specified	A high-impact mutation in the 1-deoxy-D-xylulose 5-phosphate reductoisomerase gene, affecting isoprenoid synthesis and linked to antibiotic resistance [31].
*msbA*	5059 (Genomic position on Contig CP003506)	Frameshift	Multidrug transport	Various	Mutation in the Lipid A export ATP-binding/permease protein gene, found in multiple strains, affecting a multidrug transport system [31].
Efflux ABC transporter	6855 (Genomic position on Contig CP003506)	Frameshift	Multidrug transport	Various	Unique mutation in strain S6, impacting components of an efflux ABC transporter, which can pump drugs out of the cell [31].

* 23S rRNA is a gene and therefore does not encode an amino acid change; accordingly, it is listed as ‘N/A’.

## Data Availability

No new data were created or analyzed in this study.
